# USE OF PLATELET-RICH PLASMA IN THE TREATMENT OF ROTATOR CUFF INJURIES: REVIEW OF CLINICAL TRIALS

**DOI:** 10.1590/1413-785220253304e290196

**Published:** 2025-09-08

**Authors:** João Paulo Coelho Cintra, Hugo Pinhone Ravagnani, Murillo Augusto Ribeiro do Nascimento, Henrique de Paula Rodrigues Nishimura

**Affiliations:** 1 Hospital Santa Casa de Misericordia de Ribeirao Preto, Serviço de Ortopedia e Traumatologia, Ribeirao Preto, Sao Paulo, SP, Brazil.

**Keywords:** Rotator Cuff, Platelet-Rich Plasma, Wound Healing, Regeneration, Adrenal Cortex Hormones, Manguito Rotador, Plasma Rico em Plaquetas, Cicatrização, Regeneração, Corticosteroides

## Abstract

Rotator cuff injuries occur due to overuse of the shoulder joint, trauma or age-related degeneration, and are aggravated by subacromial lesions and anatomical factors. Symptoms include persistent pain, reduced range of motion and muscle weakness, compromising patients’ quality of life. Treatment can be conservative or surgical, and platelet-rich plasma (PRP) has emerged as an alternative to speed up healing and reduce inflammation. The objective was to review the literature on the use of PRP in the treatment of rotator cuff injuries. Clinical trials published in the last 5 years and available in the PUBMED database were selected, based on the following search strategy: (platelet[title] AND rich[title] AND plasma[title]) AND (rotator[title] AND cuff[title]). The 13 studies that responded to the initial search were included in the sample that made up this review. PRP has been shown to be a promising alternative for treating rotator cuff injuries, especially in terms of short-term pain and function. However, its long-term efficacy and its comparison with corticosteroids are still a matter of debate. Therefore, the choice of treatment should be personalized, taking into account the characteristics and goals of each patient. ***Level of Evidence IV; Evidence from Descriptive (Non-Experimental) Studies or with a Qualitative Approach.***

## RESUMO

Lesões do manguito rotador ocorrem devido ao uso excessivo da articulação do ombro, traumas ou degeneração relacionada à idade, sendo agravadas por lesões subacromiais e fatores anatômicos. Os sintomas incluem dor persistente, redução da amplitude de movimento e fraqueza muscular, comprometendo a qualidade de vida dos pacientes. O tratamento pode ser conservador ou cirúrgico, e o plasma rico em plaquetas (PRP) surge como uma alternativa para acelerar a cicatrização e reduzir a inflamação. O objetivo foi revisar a literatura acerca do uso do PRP no tratamento das lesões do manguito rotador. Foram selecionados ensaios clínicos publicados nos últimos 5 anos e disponíveis na base de dados PUBMED, com base na seguinte estratégia de busca: (platelet[title] AND rich[title] AND plasma[title]) AND (rotator[title] AND cuff[title]). Os 13 estudos que responderam à busca inicial foram incluídos na amostra que constituiu a presente revisão. O PRP mostrou-se como uma alternativa promissora para tratar lesões do manguito rotador, especialmente em termos de dor e função em curto prazo. Contudo, sua eficácia em longo prazo e sua comparação com corticosteroides ainda são objeto de debate. Portanto, a escolha do tratamento deve ser personalizada, considerando as características e objetivos de cada paciente. ***Nível de Evidência IV; Evidências de Estudos Descritivos (Não Experimentais) ou com Abordagem Qualitativa.***

## INTRODUCTION

The main mechanisms that lead to injury to the rotator cuff include excessive use of the joint, such as repetitive movements, in addition to acute trauma, such as falls. Age-related degeneration is also a relevant factor, since aging especially weakens the tendons of the muscles that make up the rotator cuff. The subacromic conflict occurs when the tendons are compressed against the acromy, generating pain and inflammation. In addition, postural problems and disalignment of the shoulder can overload the tendons, while anatomical factors, such as a more pronounced acromion, can increase the predisposition to lesions.^1^

Patients with rotator cuff injury often experience persistent shoulder pain, which gets worse with daily movements and activities. The range of movement can be reduced, making it difficult to raise the arm and reach objects above the head. Muscle weakness is also common, compromising the ability to lift and carry objects, which can affect the performance of everyday tasks, such as dressing or peeling your hair. These limitations affect participation in physical and sports activities, affecting quality of life. The severity of these functional losses varies depending on the extent of the injury and the treatment received.^2^

Treatment for lesions of the rotator cuff may involve conservative and surgical approaches. Conservative measures often include physiotherapy to strengthen and improve the range of movement, use of anti-inflammatory medications to relieve pain, and adjustments in activities to prevent the injury from worsening. Corticosteroid injections can be used to reduce inflammation and pain. In turn, treatment with platelet-rich plasma (PRP) is gaining prominence in the medical environment, and is based on the use of the patient's own blood to promote healing and regeneration of injured tissues.^3^

The use of PRP involves collecting and processing the patient's blood to concentrate the platelets, which are then injected directly into the affected area. This method aims to use the growth factors present in PRP to stimulate regeneration and healing of injured tissues, taking advantage of the natural ability of the organism to promote recovery.^4^

The justification for the use of PRP in the treatment of lesions of the rotator cuff lies in its potential ability to accelerate the healing process and reduce inflammation, in addition to improving the function of the tendon. Platelets, rich in proteins and growth factors, play a crucial role in tissue regeneration, making PRP a less invasive option compared to surgery. This treatment may be especially beneficial for patients who do not respond well to traditional conservative methods, or who wish to avoid more invasive surgical procedures.

In view of the above, the proposal of this study was to review the literature about the use of PRP in the lesions of the rotator cuff, highlighting the pros and cons of this method that has been gaining space in recent years.

## MATERIAL AND METHOD

This study is set up as an integrative review of the literature, with the proposal to synthesize clinical evidence on the use of PRP in the treatment of lesions of the rotator cuff. The selection of the reviewed articles was carried out in the PUBMED database using the following search strategy: *(platelet[title] AND rich[title] AND plasma[title]) AND (rotator[title] AND cuff[title])*, including only clinical trials published in the last 5 years. The research question that guided this review was the following: "What are the effects of platelet-rich plasma when used for treatment of rotator cuff lesions?" The review process was conducted in six sequential stages, which are: a) formulation of the research question; b) identification of the relevant studies on the previously established topic; c) collection of data in the specified database; d) critical and detailed analysis of the selected studies; e) discussion of the findings of the articles; and f) elaboration and presentation of the integrative review.^5^

## RESULTS

The initial search resulted in 13 articles that met the established criteria for this survey. After reviewing the titles and summaries, all identified works were selected for review. The studies were then read in full, summarized and discussed, following a chronological order based on the year they were published. A summary of the articles selected for this review is presented in Table 1.

**Table 1:** Summary of the articles selected for this review.

Authors and Year	Original title	Type of Study	Number of Patients	Conclusion
Mohammadivahedi et al. (2024)^6^	Comparative efficacy of platelet-rich plasma (PRP) injection versus PRP combined with vitamin C injection for partial-thickness rotator cuff tears	Randomized clinical trial	110	PRP isolated and PRP with vitamin C showed similar effectiveness in reducing pain and functional improvement.
Pitsilos et al. (2024)^7^	The Biological Effect of Platelet-Rich Plasma on Rotator Cuff Tears: A Prospective Randomized In Vivo Study	Prospective randomized study	20	PRP induced beneficial microscopic changes in the healing of the supra-spinal tendon.
Rossi et al. (2024)^8^	Leukocyte-Poor Platelet-Rich Plasma as an Adjuvant to Arthroscopic Rotator Cuff Repair Reduces the Retear Rate But Does Not Improve Functional Results	Double-blind randomized clinical trial	96	PRP reduced the rate of interruption, but did not improve functional results.
Godek et al. (2022)^9^	Collagen and platelet-rich plasma in partial-thickness rotator cuff injuries	Randomized clinical trial	90	PRP and collagen showed an improvement in tendon regeneration.
Zhang et al. (2022)^10^	Injection of Leukocyte-Poor Platelet-Rich Plasma for Moderate-to-Large Rotator Cuff Tears	Prospective randomized study	104	PRP reduced relapse rate, but with no significant difference in pain and function.
Randelli et al. (2022)^11^	Platelet-Rich Plasma in Arthroscopic Rotator Cuff Repair: Clinical and Radiological Results	Randomized clinical trial	53	PRP showed no significant long-term differences.
Dyson-Hudson et al. (2022)^12^	Ultrasound-guided platelet-rich plasma injection for recalcitrant rotator cuff disease in wheelchair users	Pilot study	6	PRP was safe and reduced pain in wheelchair users.
Dadgostar et al. (2021)^13^	Corticosteroids or platelet-rich plasma injections for rotator cuff tendinopathy	Randomized clinical trial	58	PRP was more effective than corticosteroids in functional improvement.
Oudelaar et al. (2021)^14^	Efficacy of Adjuvant Application of Platelet-Rich Plasma After Needle Aspiration of Calcific Deposits	Double-blind randomized clinical trial	80	PRP showed better long-term performance than corticosteroids.
Kwong et al. (2021)^15^	Platelet-Rich Plasma in Partial-Thickness Rotator Cuff Tears Leads to Improved Short-Term Pain Relief	Randomized clinical trial	99	PRP reduced pain in the short term, but with no sustained long-term benefit.
Jo et al. (2020)^16^	Allogeneic Platelet-Rich Plasma Versus Corticosteroid Injection for Rotator Cuff Disease	Randomized clinical trial	60	PRP showed long-term functional improvements but no definitive advantage over corticosteroids.
Snow et al. (2020)^17^	The Effect of Delayed Injection of Leukocyte-Rich Platelet-Rich Plasma Following Rotator Cuff Repair	Double-blind randomized clinical trial	97	Late application of PRP did not improve function, but reduced fat infiltration.
Sari & Eroglu (2020)^18^	Comparison of ultrasound-guided PRP, prolotherapy, and corticosteroid injections in rotator cuff lesions	Randomized clinical trial	129	PRP had a longer-lasting effect than corticosteroids.

Source: Data collected by the authors.

## DISCUSSION

Mohammadivahedi et al.^6^ evaluated the ideal approach for partial treatment of rotator cuff rupture (PTRCT), which remains a controversial issue. According to the authors, recent studies have suggested that injection of platelet-rich plasma (PRP) could be a promising treatment option. However, the effects of the combined treatment of PRP and vitamin C, despite the recognized role of vitamin C in collagen synthesis and its antioxidant properties, were still not well understood. Thus, the study aimed to investigate the effect of the combined treatment of PRP and vitamin C in patients with PTRCT. For this, 110 patients were randomly assigned into two groups and sub-acromal injections of normal saline and PRP (Group A) or vitamin C and PRP (Group B) were given. Assessments were performed using the Constant score, the American Shoulder and Elbow Surgeons score (ASES) and the analogue visual scale, before and after 1 and 3 months of injections. Although both groups showed significant improvements in pain reduction and functional scores over time, there was no statistically significant difference between them. Both groups showed significant reductions in pain (p < 0.001) and increases in ASES and Constant scores (p < 0.001). Thus, it was concluded that both PRP injection alone and PRP combined with vitamin C led to significant improvements, suggesting that PRP may be effective as non-surgical treatment for PTRCTs over a 3-month period. However, it was stressed at the end of the study that additional research was still needed to determine whether the combination of PRP and vitamin C could offer significant advantages over PRP alone.

On the other hand, Pitsilos and collaborators^7^ studied the effect of PRP on tendon metabolism, which had already been extensively studied and proven *in vitro*, but whose effects *in vivo* were still little understood. A prospective, randomized, prospective study conducted by them aimed to evaluate the effect of PRP on a broken human supra-spinal tendon. To this end, 20 patients were randomized into two groups: an experimental group that received an autologous ultrasound-guided PRP injection in the subacromal space six weeks prior to scheduled surgery, and a control group that received no injections prior to surgery. The tendon samples collected during shoulder arthroscopy showed that, in the control group, there was a mixed cell population of tenocytes within disorganized collagen and accumulations of inflammatory cells. In contrast, the experimental group presented abundant oval cells with multiple cytoplasmic processes in parallel collagen fibers and less inflammation, simulating the intact structure of the tendon. These findings indicate that PRP can induce microscopic changes that stimulate the healing process and can facilitate more effective recovery.

Rossi et al.^8^, in turn, investigated whether the use of PRP as an adjuvant to the repair of the rotator cuff would improve tendon healing and functional results, a question still uncertain in clinical evidence until then. In a randomized, double-blind, controlled trial, the authors enrolled 96 patients with rotator cuff ruptures smaller than 3 cm, who were divided into two groups: a control group, submitted to double-reel suture bridge repair, and a PRP group, who received an injection of leukocyte-poor plasma (LP-PRP) during surgery. The MRI performed six months after surgery revealed that the rate of new rupture was significantly lower in the PRP group (15.2%) compared to the control group (34.1%), with a risk ratio of 0.44 (p = 0.037). However, despite the reduction in the rate of new ruptures, no significant differences in functional scores were observed between the groups. This suggests that while LP-PRP may reduce the rate of new ruptures, it did not show significant benefits in terms of postoperative pain or patients-reported outcomes.

Godek and colleagues,^9^ when investigating the lesions of the partial thick rotator cuff (PTRCI), evaluated the potential of treatments that combine collagen and PRP, collagen isolated or PRP isolated. In a study involving 90 patients conducted by the researchers, the three treatments were compared in terms of pain intensity, using a numerical classification scale, and quality of life questionnaires. Although no statistically significant differences were found between the groups, there was a trend of improvement in the groups receiving collagen with PRP or PRP isolated, suggesting that these treatments may offer benefits over collagen isolated. In addition, cases of rotator cuff regeneration have been observed in all groups, suggesting that the combined treatment of collagen and PRP may have a similar efficacy to monotherapy with collagen or PRP.

Zhang et al.^10^ discussed whether plasma rich in leukocyte-poor platelets (Lp-PRP) could reduce the rates of new rupture, decrease fat infiltration, and improve functional outcomes in patients with moderate to large degenerative rotator cuff ruptures. The study, conducted in a single center, involved 104 patients with such ruptures, who were randomly assigned into two groups: a control group, subjected only to arthroscopic repair of the double-reel suture bridge of the rotator cuff (n = 52), and a study group, who received the same repair followed by three injections of Lp-PRP at the site of tendon repair, on days 7 and 14 after surgery (n = 52). Patients were followed for an average of 27.2 months. The clinical evaluations were conducted using the UCLA scale, Constant score and analog visual scale (VAS). The integrity and fat infiltration of the repaired tissue were examined by MRI using Sugaya classification and Goutallier grade classification, 24 months after surgery. The study identified that while the UCLA and Constant and VAS mean scores showed clinically significant improvements in both groups, the differences between the groups were not statistically significant. However, the rate of new rupture was lower in the group receiving Lp-PRP (17.6%) compared to the control group (38.1%, p = 0.049). In addition, Goutallier's degree showed significant differences between the groups in the postoperative period (p = 0.03), although not in the preoperative period. The researchers concluded that repeated injections of Lp-PRP had a positive effect in reducing the rate of new rupture and improving the degree of Goutallier, providing clinical results that achieved the minimal clinically important difference for surgical treatment, although they did not show clinical improvements superior to the control group.

Randelli and collaborators,^11^ conducted a study to compare the clinical and radiological results of the arthroscopic repair of the rotator cuff with and without the addition of PRP on the tendon-bone interface after 10 years of follow-up. Of the 53 patients recruited and randomly divided into two groups (PRP = 26; control = 27), 38 were re-evaluated at least 10 years after the procedure. The clinical evaluation included multiple indices, such as UCLA score, VAS, *Simple Shoulder Test, Constant-Murley Score* (CMS), *Single Assessment Numerical Evaluation* (SANE), *American Shoulder and Elbow Surgeons* (ASES) and isometric strength. Musculoskeletal ultrasound was used to evaluate the integrity of the repaired rotator cuff. Of the 38 patients evaluated, satisfaction was high in both groups (90%), with no statistically significant differences between them. A good and excellent clinical performance was observed in both groups, but the statistical difference was noted only for ASES and SANE. The rate of new rupture was similar between the groups, with 6% in the PRP group and 14% in the control group (p = 0.61). The final conclusion of the study was that, in the long run, the clinical and radiological results were substantially uniform between the groups, and the small differences observed in 2-year follow-up did not persist.

Dyson-Hudson et al.^12^ explored the use of PRP in wheelchair users with spinal cord injury (LM) and chronic shoulder pain associated with rotator cuff disease. This pilot study aimed to test the safety and potential effect of an ultrasound-guided PRP injection for shoulder pain. Six wheelchair users with chronic shoulder pain, who failed in at least six months of conservative treatment, were given a PRP injection into the supra-spinal tendon and followed for 24 weeks. Result measures included the Wheelchair User Shoulder Pain Index (WUSPI), the Numerical Scale for Pain Assessment (NRS), physical examinations and ultrasound for supra-spinal tendinopathy, as well as the overall patient change impression (PGIC). The results showed a significant reduction in pain (WUSPI and NRS) and physical examination scores after 24 weeks. In addition, the participants reported overall improvement without adverse events or changes in ultrasound markers for tendinopathy. The researchers concluded that a single ultrasound-guided PRP injection followed by an exercise program was safe and provided improvements in pain measurements, but the lack of blindness and short-term follow-up indicate the need for a larger randomized clinical trial to confirm these findings.

Dadgostar et al.^13^ conducted a double-blind randomized clinical trial to compare the structural and clinical changes in the rotator cuff muscles after corticosteroid and PRP injections. The study included 58 patients with rotator cuff tendinitis randomly assigned to receive PRP (3 cc in the subacromyal joint and 3 cc at the site of tendon rupture) or corticosteroids (1 cc of Depo-medrol 40 mg and 1 cc of lidocaine 2%). The evaluation involved pain, range of motion (ROM), Western Ontario Rotator Cuff (WORC), Disability of Arm-Hand-Shoulder (DASH) and thickness of the supra-spinal. The results showed significant improvement in pain and ROM in both groups during follow-up. However, the PRP group showed a more significant improvement in pain and ROM, with more favorable clinical outcomes compared to the corticosteroid group. The conclusion was that PRP may be preferable to corticosteroid use due to its superior effectiveness in reducing pain and improving ROM, as well as avoiding the risks associated with corticosteroids, such as tendon rupture.

Oudelaar et al.^14^ conducted a double-blind randomized clinical trial to compare the effects of plaque-rich plasma (PRP) versus corticosteroids after calcified deposits needle aspiration (NACD) in patients with calcified rotator cuff tendinitis (RCCT). The study included 80 patients who were assigned to receive NACD followed by PRP or NACD followed by corticosteroids. The evaluation included pain, shoulder function and quality of life (QV) at various follow-up points. The NACD+PRP group was considered no inferior to the NACD+corticosteroid group in relation to the decrease in pain scores. However, the NACD+PRP group had significantly better clinical scores in the 6-month follow-up and reduced need for additional treatments, but was associated with more complications. The conclusion was that although NACD+corticosteroids showed a favorable initial effect, NACD+PRP may be an alternative with better long-term results and less need for additional treatments, despite a higher rate of complications.

Kwong et al.^15^ conducted a randomized, controlled clinical trial to compare PRP with corticosteroids in patients with partial rotator cuff rupture (PTRCTs). The study included 99 patients, and the results were evaluated based on the visual analogue scale (VAS) for pain, *American Shoulder and Elbow Surgeons* (ASES) and *Western Ontario Rotator Cuff Index* (WORC). The PRP group showed a superior improvement in pain and function compared to the corticosteroid group in the 3-month follow-up. However, there were no sustained differences in long-term follow-up (12 months), and the rate of failure and conversion to surgery was similar between the groups. The conclusion was that while PRP provided superior improvement in short-term pain and function, there was no sustained long-term benefit compared to corticosteroids.

Jo et al.^16^ conducted a randomized controlled clinical trial to investigate the efficacy and safety of allogenic pure PRP injections compared to corticosteroids for the treatment of rotator cuff disease. The study included 60 patients, who were randomly assigned to receive a subacromal injection of pure allogenic PRP or a mixture of corticosteroid and lidocaine. The primary outcomes were safety and Constant score in 1 month, while the secondary outcomes included pain, amplitude of movement, muscle strength and satisfaction. There were no adverse events related to treatment. Although the Constant score at 1 month showed no significant differences between the groups, the PRP group showed significant improvements in DASH score, overall function and external rotation at 6 months. Shoulder pain and function improved slowly in the PRP group, while the corticosteroid group showed a rapid response but no further long-term improvements. The conclusion was that while PRP is safe and can offer functional benefits in terms of overall function and external rotation, it has not been shown to be definitely superior to the corticosteroid in terms of pain relief and functional improvement.

Snow et al.^17^ investigated the impact of late application of leukocyte-rich PRP on the repair of the rotator cuff. The study included 97 patients with symptomatic rotator cuff rupture who were randomized to receive a PRP or normal saline solution injection between 10 and 14 days after surgery. The evaluation included patient-reported outcome scores and MRI images to evaluate the integrity of repair and fat infiltration. In 1 year, there were no significant differences between the groups in outcome scores or new break rates. However, fat infiltration was significantly lower in the PRP group compared to the control group. The conclusion was that the late application of post-repair rotator cuff PRP did not improve the function as measured by the patients’ reported outcomes and Constant score, but may have a positive effect on reducing fat infiltration.

These studies suggest that while PRP may offer benefits in some aspects, such as reducing fat infiltration and improvements in certain functional parameters, its effectiveness compared to corticosteroids may vary. The decision on the use of PRP versus corticosteroids should consider both the potential benefits and clinical outcomes observed in specific studies.

Finally, Sari and Eroglu^18^ conducted a study to compare the efficacy of different injection methods for rotator cuff lesions, including PRP, corticosteroids and prolotherapy. The study involved 129 patients, divided into four groups: PRP, corticosteroid (COR), prolotherapy (PRO) and lidocaine. All patients received a subacromal injection and were evaluated using the Visual Analogous Scale (VAS), the Standardized Shoulder Assessment Form of the American Shoulder and Claw Surgeons (ASES) and the Western Ontario Rotator Cuff Index (WORC) at 3, 12 and 24 weeks after injection. The results indicated that at week 3, the corticosteroid group showed a significant reduction in pain (VAS) and a improvement in function (WORC) compared with the other groups, demonstrating a faster relief. However, over 24 weeks, the PRP group surpassed the corticosteroid group in terms of reducing pain and improving function, suggesting that PRP could provide more lasting benefits. In addition, the corticosteroid group had a significantly higher ASES score than the PRP and PRO groups at week 3, showing a faster improvement in function. In short, corticosteroid injection provides faster pain relief and short-term function improvement, while PRP stands out for its long-lasting and lasting benefits. All evaluated injection methods improved patient pain, function and quality of life, but PRP proved to be the most effective option for long-term well-being.

The analysis of the studies selected for this review revealed important convergences and divergences between the studies. In general, there is consensus that PRP provides a significant improvement in patient pain, especially in the short term,^13,15,18^ in addition to contributing to the improvement of shoulder function and amplitude of movement. Some studies also indicate that this therapeutic approach reduces the rates of new tendon rupture when used as an adjuvant to surgery, suggesting a positive role in tissue healing.^8,10^ Furthermore, the safety of therapy was reiterated by several authors, who did not report serious adverse events related to the use of PRP.^12,16^

Despite these agreements, there are still major differences as to the long-term effectiveness of the PRP. While some studies point to sustained benefits,^11^ others did not identify significant differences between PRP and corticosteroids after a prolonged period of follow-up.^17^ The comparison between these two therapies, by the way, also produced different results. Some studies also suggest that PRP provides longer functional recovery,^13,14^ while others indicate that corticosteroids provide faster pain relief, but without a prolonged effect.^15,16^

Another issue discussed was the combination of PRP with other substances, such as vitamin C. Although this association has been tested, it did not demonstrate additional advantages over PRP alone.^6^ Furthermore, the analysed studies showed variations in application protocols, including differences in the amount, frequency and time ideal for administration, which impacts the standardization of the technique and makes direct comparisons between the results difficult.^10,17^

Overall, there has been growing consensus on the efficacy of PRP in the treatment of rotator cuff lesions, especially for reducing pain and improving function in the short term. PRP has proven to be a promising alternative to corticosteroids, offering notable clinical benefits, such as reducing pain and improving movement amplitude. However, its efficacy combined with other substances, such as vitamin C, is still debated, with studies showing that the combination showed no significant advantages over PRP alone. In addition, while PRP can contribute to tendon healing and reduction of fatty infiltration, its long-term effects and compared to corticosteroids are varied, with some studies indicating that corticosteroids offer faster relief, while PRP can provide lasting benefits. The choice of treatment should therefore consider the individual characteristics of the patients and the specific goals of the treatment.

## CONCLUSION

PRP represents a promising alternative for the treatment of rotator cuff lesions, especially for pain relief and short-term functional improvement. However, its long-term effectiveness and its comparison with other therapies still require further studies. The choice of PRP should take into account the particularities of each patient, the severity of the injury, and the goals expected with the treatment, in order to ensure the best possible therapeutic approach.
